# Association of the Chinese visceral adiposity index with marrow adiposity in postmenopausal females

**DOI:** 10.3389/fendo.2025.1542516

**Published:** 2025-02-25

**Authors:** Xiaoyong Zuo, Zeyang Miao, Run Xu, Dan Shi, Shixin Chang, Guanwu Li, Peng Luo

**Affiliations:** ^1^ Department of Radiology, Yueyang Hospital of Integrated Traditional Chinese and Western Medicine, Shanghai University of Traditional Chinese Medicine, Shanghai, China; ^2^ Department of Geriatrics, Yueyang Hospital of Integrated Traditional Chinese and Western Medicine, Shanghai University of Traditional Chinese Medicine, Shanghai, China

**Keywords:** marrow adipose tissue, proton density fat fraction, Chinese visceral adiposity index, bone mineral density, menopause

## Abstract

**Objective:**

To investigate the relationship between the Chinese visceral adiposity index (CVAI) and vertebral proton density fat fraction (PDFF).

**Methods:**

The study included 181 postmenopausal females including 53 normal bone mineral density (BMD), 88 osteopenia, and 40 osteoporosis. Vertebral marrow PDFF was measured using Fat Analysis & Calculation Technique imaging, and BMD was assessed via dual-energy X-ray absorptiometry. Bone turnover biomarkers and lipid metabolism were evaluated. The relationship between CVAI and PDFF was assessed using multivariable linear regression models, generalized additive models, and threshold effect analysis.

**Results:**

The mean BMD at the lumbar spine increased, and PDFF significantly decreased as quartiles of CVAI increased (*P* for trends <0.05). Multivariable linear regression analysis revealed a clear negative correlation between CVAI and PDFF (regression coefficient β = −0.251, 95% CI, −0.303 to −0.200; *P* < 0.001) after adjusting for age, time since menopause, waist circumference, body mass index, physical activity, and lipid profiles. The association with marrow PDFF remained significant (β = −0.202, 95% CI, −0.255 to −0.149, *P* < 0.001) even after additional adjustment for BMD. Further analysis revealed an L-shaped non-linear relationship between CVAI and marrow PDFF after adjusting for age, time since menopause, waist circumference, body mass index, physical activity, lipid profiles, and BMD. An inflection point was identified at a CVAI of 128.3, below which each one-unit increase in CVAI corresponded to a more substantial decrease in marrow PDFF (β = −0.0055, 95% CI: −0.0064 to −0.0045; *P* < 0.001). However, above this inflection point, each unit increase in CVAI was not significantly associated with a decrease in marrow PDFF.

**Conclusions:**

CVAI exhibited a nonlinear negative association with marrow adiposity within a suitable range, once CVAI crossed a definite threshold, PDFF ceased to increase. This finding suggests that a moderate visceral fat accumulation may enhance skeletal integrity, while excessive visceral fat could potentially have detrimental effects.

## 1 Introduction

The visceral adiposity index (VAI) has emerged as a reliable indicator for visceral adiposity. VAI has shown notable correlations with various metabolic disorders, including osteoporosis, diabetes mellitus, heart failure, cerebrovascular diseases, polycystic ovary syndrome, and metabolic fatty liver disease/non-alcoholic steatohepatitis (1–5). However, the correlation between VAI and adipose tissue area has been modest among Chinese populations, highlighting significant diversity in body fat distribution across ethnic groups (6). The Chinese visceral adiposity index (CVAI), incorporating age, body mass index (BMI), waist circumference, triglycerides (TG), and high-density lipoprotein cholesterol (HDL-c), has been validated for assessing visceral fat dysfunction. In a study of 6495 Chinese individuals, CVAI demonstrated a strong correlation between visceral obesity and insulin resistance, outperforming BMI and waist circumference in identifying metabolic conditions. The area under the receiver operating characteristic curve for visceral obesity (0.83) significantly exceeded that for VAI (0.69) (6).

The relationship between the VAI and bone mineral density (BMD) is inconsistent. Some studies suggest a positive correlation, indicating that higher VAI is linked to a lower prevalence of osteoporosis (4, 7–9), while others identify VAI as an independent predictor of trabecular bone loss (10). The discrepancies in understanding the relationship between obesity indices and osteoporosis stem, in part, from variations in obesity classification and the intricate, nonlinear dynamics of these associations. Moreover, the influence of obesity and adiposity on osteoporosis manifests differently in men and women (11). Consequently, a more comprehensive investigation into the connection between the VAI and bone integrity is imperative.

Marrow adipocytes reside in the bone marrow alongside bone and hematopoietic cells. Despite the growing recognition of marrow adipocytes as a therapeutic target, much remains to be understood about their roles in metabolism, skeletal homeostasis, cancer, and regenerative treatments (12). Expansion of marrow fat is observed in conditions such as osteoporosis, obesity, diabetes, and even unexpectedly, anorexia nervosa (13–15). This increase in marrow adipose tissue often correlates with a decrease in bone mass, thereby increasing the risk of fractures, as evidenced by several studies of negative correlation between vertebral marrow proton density fat fraction (PDFF) and BMD, although the causal relationship in this association remains unclear.

Previous studies have predominantly explored the linear relationship between marrow adiposity and factors such as BMI, total fat mass, as well as visceral and subcutaneous adipose tissue (16–20). However, research directly examining the association between VAI, particularly the CVAI, and marrow adiposity in humans remains limited. Considering that VAI has been linked to BMD, and BMD is also associated with marrow adiposity, it is reasonable to hypothesize a potential connection between VAI and marrow adiposity. Therefore, the primary objective of this study is to explore the non-linear association between CVAI and PDFF using a three-dimensional Fat Analysis & Calculation Technique sequence, with a particular focus on postmenopausal women.

## 2 Methods

### 2.1 Study participants

In this cross-sectional study, we examined a cohort of postmenopausal females (n = 181) aged between 50 and 87 years between March 2019 and June 2023, who self-reported cessation of menstruation for more than 1 year. Menopause was defined as the absence of menstruation for at least 12 months following the last menstrual period (21). Exclusion criteria included: 1) Use of medications known to affect bone density and potentially bone marrow lipids, including glucocorticoids, osteoporosis medications such as bisphosphonates, raloxifene, calcitonin, or teriparatide, as well as hormone therapy involving estrogen and testosterone; 2) Medical conditions altering bone metabolism, such as diabetes mellitus, malignancies, hypo- or hyperparathyroidism, hypo- or hyperthyroidism, chronic hepatic or renal diseases, multiple myeloma, and rheumatoid arthritis; 3) Any potential confounding factor capable of influencing the interpretation of results, such as severe lumbar spine deformity.

A standardized physical examination was conducted for all participants, recording data on age, menopausal status, waist circumference, body weight, height, and physical activity. Physical activity was defined as engaging in moderate exercise at least three times per week for a minimum of 30 minutes per session (22). BMI was then computed by dividing weight in kilograms by height in meters squared. Anthropometric data, including BMI and waist circumference, and biochemical indicators such as TG and HDL-c, were utilized to calculate CVAI. The CVAI for females was calculated using the following formula: CVAI = −187.32 + 1.71 × age + 4.23 × BMI + 1.12 × waist circumference (cm) + 39.76 × Log10(TG) - 11.66 × HDL-c (6). Waist circumference and BMI were measured in centimeters and kilograms per square meter respectively, while TG and HDL-c were recorded in millimoles per liter.

The research protocol obtained approval from the institute’s Ethics Committee, ensuring compliance with the ethical principles delineated in the Declaration of Helsinki and its subsequent revisions. Before they participated in the study, all participants provided written informed consent.

### 2.2 Laboratory analyses

Morning blood samples for laboratory analysis were obtained from all participants between 7:00 and 10:00 AM following an 8-hour overnight fast. Plasma fasting glucose and lipid profiles (including TG, total cholesterol, HDL-c, and low-density lipoprotein cholesterol) were measured on the same collection day using a chemiluminescence immunoassay system analyzer. Serum levels of bone turnover biomarkers, such as 25-hydroxyvitamin D, N-terminal propeptide of type 1 procollagen, β-type I collagen telopeptides, and parathyroid hormone, were assessed using an electrochemiluminescence immunoassay system analyzer (Cobas 8000 e801; Roche Diagnostics, Basel, Switzerland).

### 2.3 BMD measurements

Area BMD (g/cm^2^) was measured using dual-energy X-ray absorptiometry (Prodigy Lunar; GE Healthcare, Waukesha, WI). BMD assessments were performed at the lumbar spine (L1-L4) and left hip. The diagnosis was established by a single consultant using T-score values according to the criteria outlined by the World Health Organization. Daily quality control for dual-energy X-ray absorptiometry was carefully supervised by the same technician to ensure consistency and maintain variation levels below 2%.

### 2.4 MR image acquisition and data analysis

All MRI scans were performed using a clinical 3T MRI system (uMR 780, United Imaging Healthcare, Shanghai, China) on the same day as the dual-energy x-ray absorptiometry scan. To identify any pre-existing abnormalities of the lumbar vertebrae, routine MR imaging of the lumbar spine was conducted using sagittal T1-weighted and T2-weighted sequences, along with a transverse T2-weighted sequence. Subsequently, a six-echo 3D spoiled gradient-echo sequence, known as the 3D Fat Analysis & Calculation Technique sequence, was utilized to achieve chemical shift-encoded water-fat separation in the lumbar spine. For specific imaging parameters, please refer to the detailed descriptions in previous literature (13, 23).

The gradient echo imaging data underwent real-time processing using the fat quantification routine provided by the vendor. This routine initially conducts phase error correction and then proceeds with complex-based water-fat decomposition. It utilizes a pre-calibrated seven-peak fat spectrum and a single T2* to model signal variation with echo time, ultimately generating PDFF and R2* maps. Vertebral bone marrow PDFF was determined by averaging measurements from four positioned regions of interest (ROIs) using a postprocessing workstation (uWS-MR Advanced Postprocess Workstation, United Imaging Healthcare). These ROIs were meticulously positioned within the bodies of the L1 to L4 vertebrae, spanning three-fourths of their height, with careful exclusion of cortical bone and endplates. This process was independently conducted by two raters. Subsequently, the PDFF values were computed by averaging the measurements obtained from the four vertebrae.

### 2.5 Statistical analysis

Continuous variables with a normal distribution were presented as mean (standard deviation, [SD]), while skewed variables were reported as median (interquartile range, [IQR]). Categorical variables were represented as frequencies (%). The clinical characteristics of participants with varying bone density and across CVAI quartiles were compared using one-way ANOVA, Kruskal-Wallis, and chi-square tests, as appropriate, with multiple comparisons adjusted using the Bonferroni correction. Differences in CVAI and PDFF among different bone mass groups were tested using analysis of covariance. We utilized a polynomial test to examine the trends in PDFF and BMD across CVAI quartiles. After adjusting for age, time since menopause, waist circumference, BMI, physical activity, lipid profiles, and BMD, we compared the least square mean value of BMD and PDFF across quartiles of CVAI. Subsequently, a multiple linear regression analysis was performed to evaluate the presence of a linear trend. Pearson’s and Spearman’s correlation analyses were conducted to examine bivariate relationships between CVAI, marrow PDFF, and BMD, accounting for normal and non-normal data distributions where applicable. Linear regression models were employed to investigate the associations between CVAI and marrow PDFF, yielding regression coefficients (β) and their corresponding 95% confidence intervals (CI).

To explore the complex, non-linear relationship between CVAI and marrow PDFF, we employed the Generalized Additive Model (GAM). This model is particularly well-suited for examining nonlinear associations without assuming parametric forms. By applying nonparametric smoothing functions, GAM offers a more flexible approach to understanding how predictors influence the response variable. The implementation of the GAM involved defining the response distribution, specifying the mean model, and using iterative algorithms for parameter estimation. This allowed for both model evaluation and statistical inference (24). To further investigate threshold effects within the smoothing curves, we applied a two-piecewise linear regression model, which was enhanced by a recursive method to identify inflection points. This combination of methods provided deeper insights into the underlying nonlinearity of the data. All analyses were conducted using the statistical software packages R-4.4.1 and SPSS (version 27.0; Chicago, IL, USA), and a two-tailed *p*-value of less than 0.05 was considered to signify statistical significance.

## 3 Results

### 3.1 Clinical characteristics

**Table 1** summarizes the basic characteristics of the study population. Three age-matched groups were established based on dual-energy X-ray absorptiometry T-score results: 53 individuals with normal BMD, 88 with osteopenia, and 40 with osteoporosis. No significant differences were observed among the three groups in terms of physical activity, body height, 25-hydroxyvitamin D, N-terminal propeptide of type 1 procollagen, β-type I collagen telopeptides, osteocalcin, parathyroid hormone, and lipid profiles. However, differences were noted among the groups in time since menopause, body weight, waist circumference, and BMI (*P* < 0.05) (**Table 1**).

**Table 1:** Baseline characteristics of the participants with varying bone density.

Variables	Normal bone mass (n =53)	Osteopenia (n =88)	Osteoporosis (n =40)
Age, years	66.5 ± 4.8	68.9 ± 6.7	69.1 ± 5.1
Time since menopause, years	16.1 ± 5.4	18.9 ± 7.6 ^a^	19.2 ± 6.0
Physical activity, n (%)	16 (30.2%)	31 (35.2%)	14 (35.0%)
Body weight, kg	63.4 ± 7.2	57.1 ± 5.8 ^a^	56.6 ± 6.4 ^a^
Height, cm	159.0 ± 4.7	158.0 ± 5.2	157.0 ± 5.3
Body mass index, kg/m^2^	25.2 ± 2.9	22.9 ± 2.0 ^a^	23.1 ± 2.6 ^a^
Waist circumference, cm	78.3 ± 14.7	71.5 ± 9.2 ^a^	72.3 ± 11.2 ^a^
25-hydroxyvitamin D, nmol/L	57.9 (44.3, 65.7)	56.3 (47.4, 75.1)	53.8 (39.8, 74.8)
P1NP, ng/mL	48.0 (38.0, 66.9)	56.0 (44.8, 69.5)	50.7 (40.9, 65.0)
β-type I collagen telopeptides, pg/mL	421 (323, 584)	491 (361, 656)	469 (361, 615)
Osteocalcin, ng/mL	19.3 (15.3, 23.5)	19.5 (16.7, 25.3)	18.4 (15.7, 24.3)
Parathyroid hormone, pg/mL	4.74 (3.27, 5.85)	4.23 (3.25, 5.57)	3.71 (3.09, 4.88)
Lumbar spine BMD, g/cm^2^	1.199 ± 0.117	0.941 ± 0.076 ^a^	0.820 ± 0.144 ^ab^
Femoral neck BMD, g/cm^2^	0.915 ± 0.102	0.759 ± 0.089 ^a^	0.693 ± 0.103 ^ab^
Total hip BMD, g/cm^2^	0.976 ± 0.099	0.815 ± 0.079 ^a^	0.748 ± 0.090 ^ab^
Triglycerides, mmol/L	1.39 (1.23, 1.65)	1.38 (1.08, 1.72)	1.29 (0.91, 1.76)
Total cholesterol, mmol/L	5.62 ± 1.09	5.24 ± 1.00	5.85 ± 1.27
HDL_c, mmol/L	1.46 (1.31, 1.75)	1.54 (1.35, 1.70)	1.68 (1.38, 1.81)
LDL_c, mmol/L	3.46 (3.09, 3.93)	3.52 (2.76, 3.87)	3.50 (2.93, 4.42)

Data were expressed as mean ± SD, median (interquartile range) or n (%), where appropriate.

BMD, bone mineral density; P1NP, N-terminal propeptide of type 1 procollagen; HDL-C, high-density lipoprotein cholesterol; LDL-C, low-density lipoprotein cholesterol.

^a^ *P*<0.05 vs. normal bone mass and ^b^ *P*<0.05 vs. osteopenia.

Significance values have been adjusted by the Bonferroni correction for multiple tests.

The cohort distribution based on CVAI was divided into quartiles as follows: quartile 1, ≤80.5; quartile 2, 80.6 ≤ Q2 ≤ 95.0; quartile 3, 95.1 ≤ Q3 ≤ 121.0; and quartile 4, ≥121.1. Across the various CVAI quartiles (Q1-Q4), significant differences were observed in age, time since menopause, body weight, waist circumference, BMI, TG, and HDL-c, as shown in **Table 2**.

**Table 2:** Baseline characteristics of the participants across CVAI quartiles.

Variables	CVAI Q1≤80.5 (n =45)	80.6≤Q2≤95.0 (n =48)	95.1≤Q3≤121.0 (n =44)	Q4≥121.1 (n =44)
Age, years	64.6 ± 4.4	68.4 ± 5.6 ^a^	70.2 ± 7.3 ^a^	69.8 ± 6.0 ^a^
Years since menopause, years	14.2 ± 4.9	18.2 ± 6.0	20.5 ± 7.3 ^a^	19.8 ± 7.0 ^a^
Physical activity, n (%)	11 (24.4%)	15 (31.3%)	18 (40.9%)	17 (38.6%)
Body weight, kg	53.7 ± 4.7	56.0 ± 5.1	59.8 ± 5.6 ^ab^	66.1 ± 5.6 ^abc^
Height, cm	158.0 ± 4.9	159.0 ± 5.8	158.0 ± 5.2	157.0 ± 4.4
Body mass index, kg/m^2^	21.5 ± 1.17	22.2 ± 1.4	24.0 ± 1.7 ^ab^	27.0 ± 2.1 ^abc^
Waist circumference, cm	66.1 ± 3.2	67.7 ± 5.0	76.8 ± 7.1^ab^	91.3± 9.3^abc^
25-hydroxyvitamin D, nmol/L	51.6 (40.6, 71.8)	59.8 (48.5, 74.8)	55.3 (46.8, 69.9)	58.3(46.7, 72.5)
P1NP, ng/mL	49.6 (41.2, 65.8)	51.7 (39.2, 69.6)	59.2 (45.4, 69.5)	53.5(40.4, 68.3)
β-type I collagen telopeptides, pg/mL	493 (380, 640)	442 (319, 602)	428 (330, 606)	486(393, 641)
Osteocalcin, ng/mL	19.9 (16.9, 25.3)	18.7 (14.8, 22.5)	20.0 (16.1, 25.9)	18.9(16.2, 22.0)
Parathyroid hormone, pg/mL	3.84 (3.34, 5.74)	4.31 (3.13, 5.15)	4.39 (3.10, 6.36)	4.21(3.37, 5.39)
Lumbar spine BMD, g/cm^2^	0.934 ± 0.160	0.960 ± 0.149	0.979 ± 0.164	1.090 ± 0.200 ^abc^
Femoral neck BMD, g/cm^2^	0.768 ± 0.113	0.789 ± 0.113	0.771 ± 0.135	0.833 ± 0.141
Total hip BMD, g/cm^2^	0.808 ± 0.115	0.849 ± 0.102	0.827 ± 0.119	0.905 ± 0.139^ac^
Triglycerides, mmol/L	1.01 (0.86, 1.27)	1.48 (1.18, 2.05)^a^	1.37 (1.19, 1.72)^a^	1.60(1.36, 2.06)^a^
Total cholesterol, mmol/L	5.84 ± 1.17	5.51 ± 1.24	5.59 ± 0.90	5.37 ± 1.01
HDL_c, mmol/L	1.70 (1.61, 1.91)	1.47 (1.32, 1.71)^a^	1.54 (1.35, 1.75)^a^	1.39 (1.28, 1.54)^a^
LDL_c, mmol/L	3.56 (2.94, 4.20)	3.40 (2.63, 4.12)	3.51 (3.16, 3.82)	3.48 (2.83, 3.86)

Data were expressed as mean ± SD, median (interquartile range) or n (%), where appropriate.

BMD, bone mineral density; P1NP, N-terminal propeptide of type 1 procollagen; HDL-C, high-density lipoprotein cholesterol; LDL-C, low-density lipoprotein cholesterol; Q, Quartile.

^a^ *P*<0.05 vs. Q1, ^b^ *P*<0.05 vs. Q2, and ^c^ *P*<0.05 vs. Q4.

Significance values have been adjusted by the Bonferroni correction for multiple tests.

### 3.2 Relationships between CVAI, marrow fat fraction, and BMD

**Figure 1:**
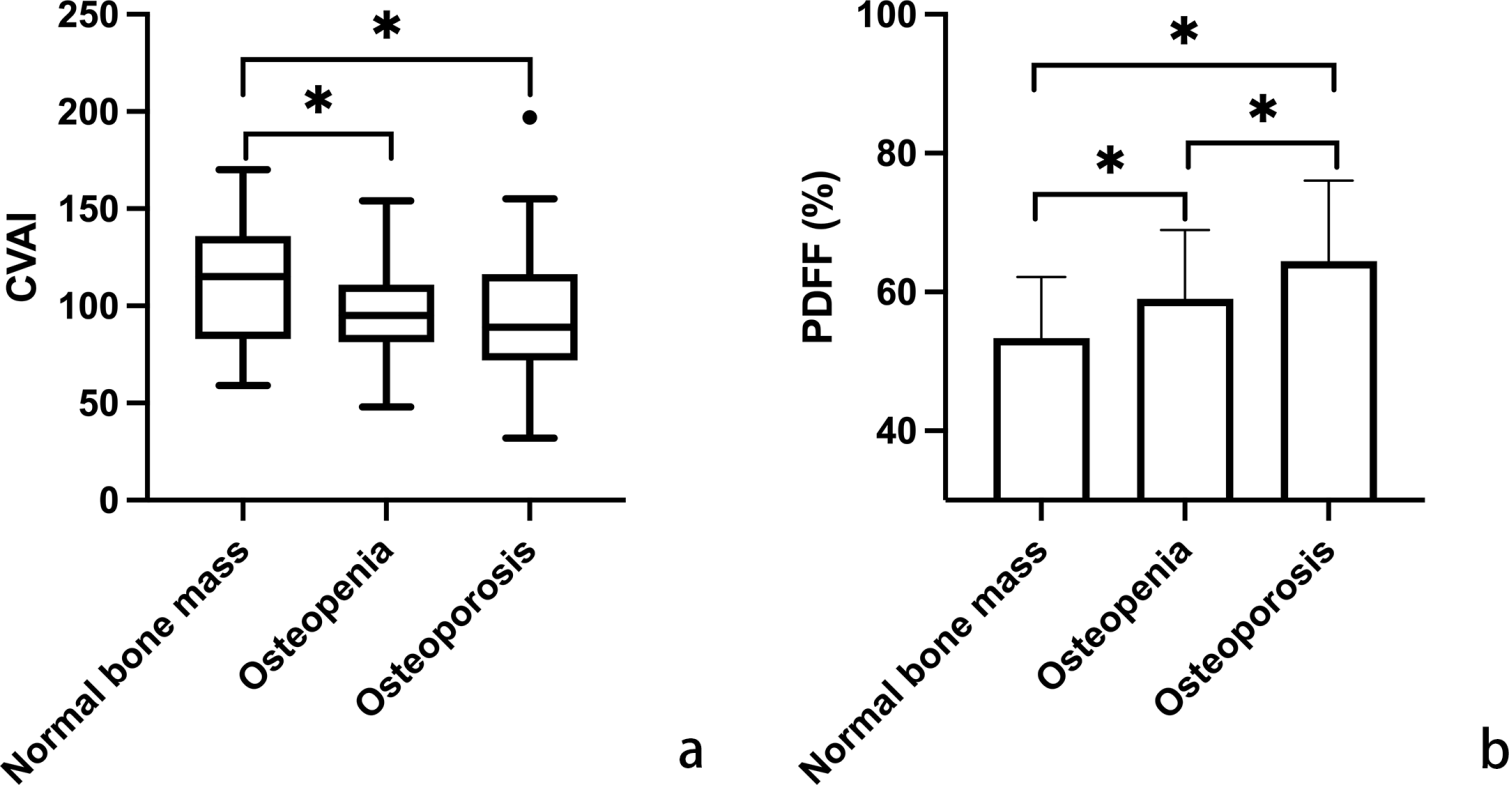
Comparative analysis of Chinese visceral adiposity index (CVAI) (**a**; using box plot for non-normal distribution) and marrow proton density fat fraction (PDFF) (**b**; using histogram for normal distribution) across the categories of normal bone mass, osteopenia, and osteoporosis. Horizontal lines indicate significant differences between groups, evaluated by *post hoc* analysis with Tukey-adjusted p-values for distinguishing different bone mineral density T-scores (*p < 0.05). CVAI is real numbers without any unit.

**Figure 2:**
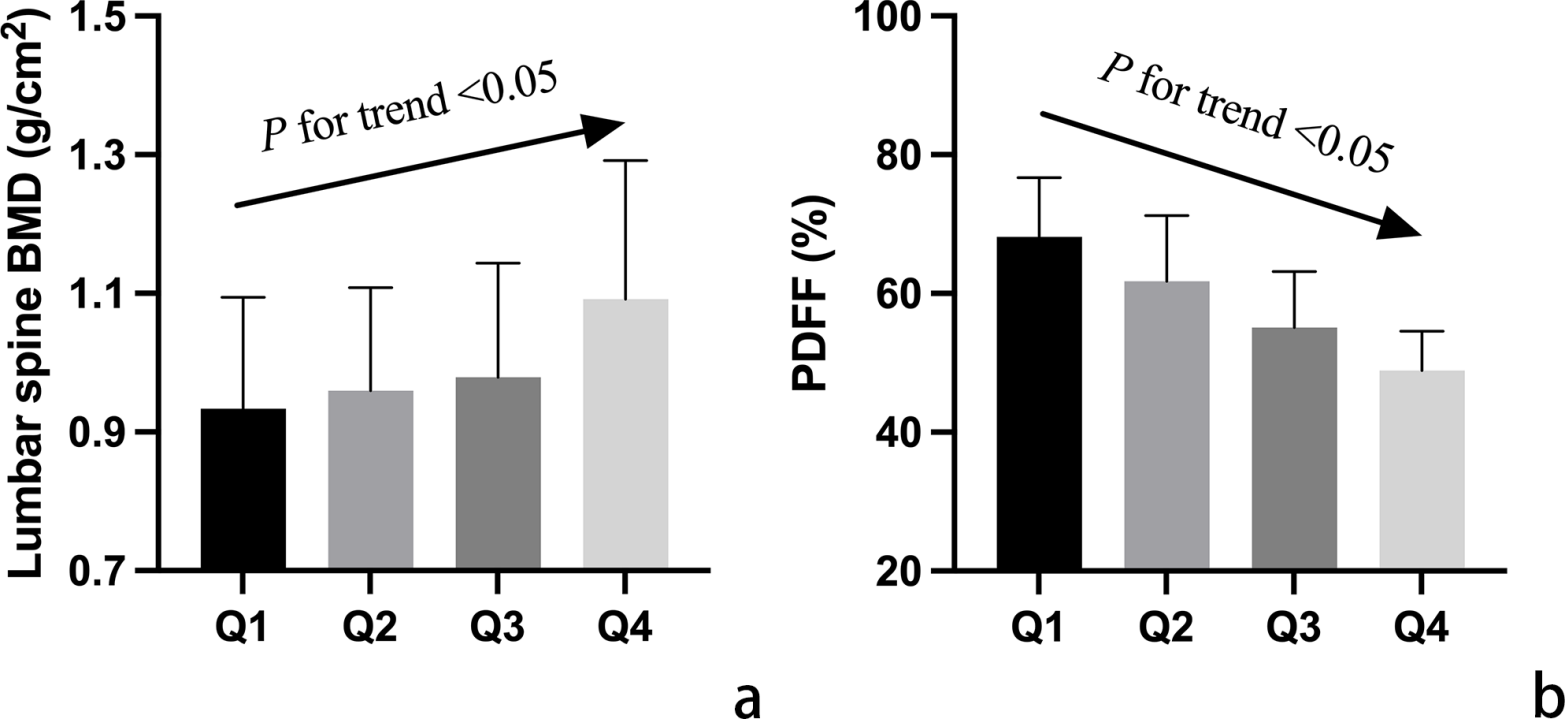
Adjusted mean values of vertebral bone mineral density (BMD) **(a)** and marrow proton density fat fraction (PDFF) **(b)** among participants categorized into quartiles (Q1-Q4) based on Chinese visceral adiposity index (CVAI). Mean values were adjusted for age, time since menopause, waist circumference, body mass index, physical activity, and lipid profiles. The arrow signifies a notable linear correlation between CVAI quartile groups and both lumbar spine BMD and PDFF values.

In **Figure 1**, variations in CVAI and PDFF among individuals with normal bone mass, osteopenia, and osteoporosis are depicted. Analysis results indicated that individuals with osteopenia and osteoporosis displayed significantly decreased CVAI and elevated PDFF levels compared to those with normal bone mass (*P* < 0.05). The least square means of lumbar spine BMD and vertebral PDFF were compared across quartiles of CVAI after adjusting for age, time since menopause, waist circumference, BMI, physical activity, and lipid profiles. As illustrated in **Figure 2**, higher CVAI quartiles were associated with elevated BMD and significantly decreased PDFF compared to lower quartiles (*P* for trend < 0.05 for all).

Based on the correlation analysis results (**Table 3**), a positive correlation emerged between CVAI and BMD at the lumbar spine (*r*= 0.336, *P*<0.001), femoral neck (*r*= 0.184, *P*= 0.013), and total hip (*r*= 0.253, *P*<0.001). Conversely, a negative correlation was observed between CVAI and PDFF (*r*= −0.661, *P*<0.001), as well as between BMD and PDFF(*r*= −0.234 to −0.480, all *P*<0.01).

**Table 3:** Correlation coefficients among the CVAI, marrow PDFF, and BMD.

		CVAI	PDFF	Lumbar spine BMD	Femoral neck BMD	Total hip BMD
CVAI	*r*	1.000	−0.661	0.336	0.184	0.253
	*p-value*		<0.001	<0.001	0.013	<0.001
PDFF	*r*	−0.661	1.000	−0.480	−0.234	−0.293
	*p-value*	<0.001		<0.001	0.002	<0.001
Lumbar spine BMD	*r*	0.336	-0.480	1.000	0.534	0.623
	*p-value*	<0.001	<0.001		<0.001	<0.001
Femoral neck BMD	*r*	0.184	−0.234	0.534	1.000	0.902
	*p-value*	0.013	0.002	<0.001		<0.001
Total hip BMD	*r*	0.253	−0.293	0.623	0.902	1.000
	*p-value*	<0.001	<0.001	<0.001	<0.001	

BMD, bone mineral density; CVAI, Chinese visceral adiposity index; PDFF, proton density fat fraction.

### 3.3 Multivariate regression analysis

Linear regression analysis demonstrated that CVAI independently served as a positive predictor of lumbar spine BMD, with a regression coefficient of ß = 0.686, 95% CI, 0.438 to 0.934, *P <*0.001. To assess the independent relationship between CVAI and PDFF, a multiple linear regression model was employed. CVAI significantly influenced vertebral PDFF in the multiple regression analysis (regression coefficient ß = −0.251, 95% CI, −0.303 to −0.200; *P* < 0.001), controlling for age, time since menopause, waist circumference, BMI, physical activity, and lipid profiles. This association remained statistically significant in the multivariate linear regression analysis even after additional adjustment for BMD (regression coefficient ß = −0.202, 95% CI, −0.255 to −0.149, *P* < 0.001).

### 3.4 Nonlinear relationship exploration between CVAI and PDFF

**Figure 3:**
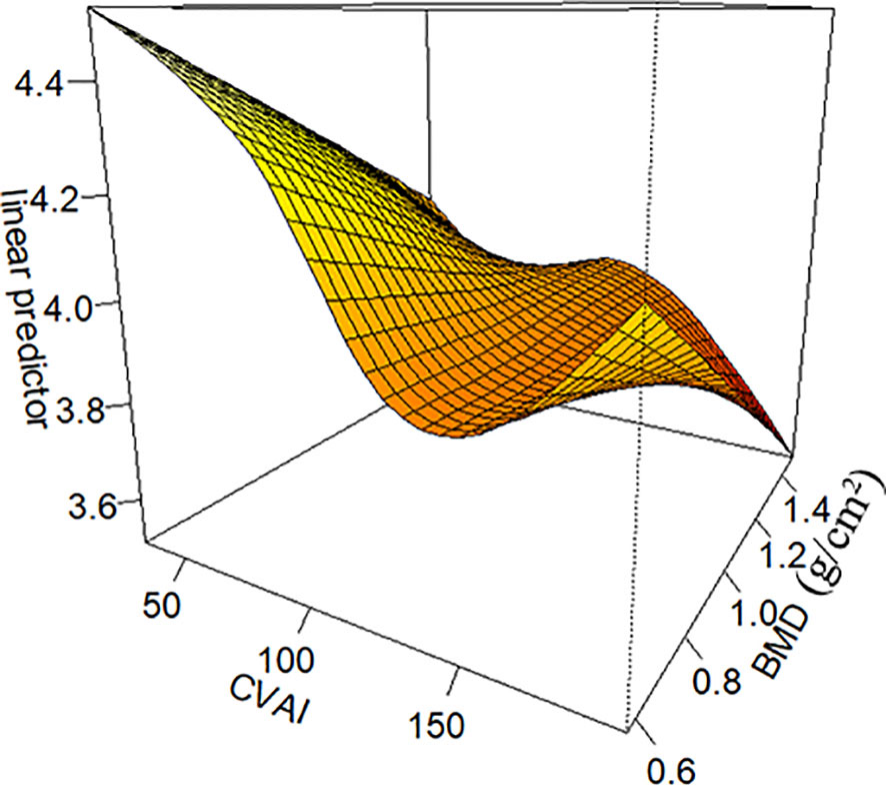
The interaction effects between Chinese visceral adiposity index (CVAI) and lumbar spine bone mineral density (BMD). CVAI exerts an interactive effect with BMD, as illustrated in **Figure 3**. The impact of CVAI on bone marrow PDFF varies depending on BMD levels. At low bone mass, both excessively low and high CVAI are associated with increased PDFF, with the former causing a more significant rise. In contrast, at higher bone mass and higher CVAI, the bone marrow PDFF is observed to be at its lowest. CVAI is real numbers without any unit.

**Figure 4:**
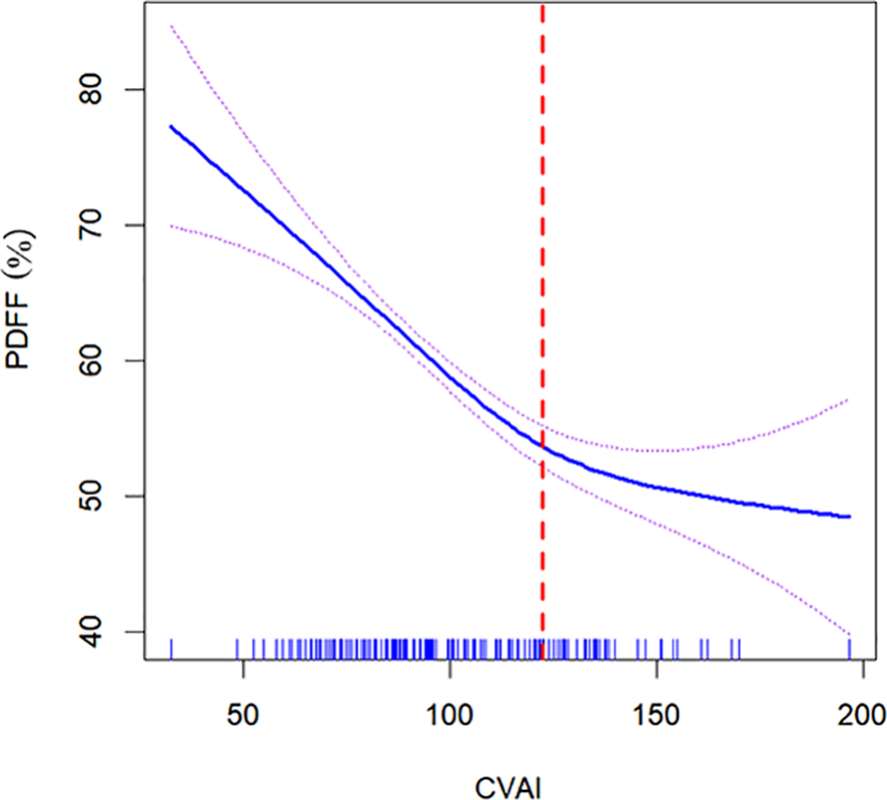
Nonlinear relationship of Chinese visceral adiposity index (CVAI) and lumbar spine proton density fat fraction (PDFF). The solid blue line in the middle represents a smooth curve fitting between the variables. The dotted lines on both sides indicate the 95% confidence interval (CI) for the fit. An inflection point at a CVAI of 128.3 (red dotted line) shows a likelihood ratio test *P* value of <0.001, indicating statistical significance. The *P* value for non-linearity is <0.001. The model was adjusted for age, time since menopause, waist circumference, body mass index, physical activity, lipid profiles, and bone mineral density. CVAI is real numbers without any unit.

To investigate the non-linear relationship between CVAI and PDFF, we employed a generalized additive model alongside a smooth curve fitting analysis. The interaction effects between CVAI and BMD are shown in **Figure 3**. **Figure 4** reveals an L-shaped non-linear association between CVAI and PDFF after adjustments for age, time since menopause, waist circumference, BMI, physical activity, lipid profiles, and BMD. Additionally, a threshold effect analysis, utilizing a two-segment linear regression model, identified an inflection point at CVAI = 128.3. Below this threshold, each unit increase in CVAI corresponds to a 0.0055 decrease in vertebral PDFF (β = −0.0055, 95% CI: −0.0064 to −0.0045; *P* < 0.001). In contrast, above the inflection point, each unit increase in CVAI is not significantly associated with a decrease in vertebral PDFF, which is 0.0006 (β = −0.0006, 95% CI: −0.0030 to 0.0018; *P* = 0.618) (**Table 4**).

**Table 4:** Threshold effect analysis of the relationship of Chinese visceral adiposity index (CVAI) with marrow proton density fat fraction (PDFF).

	Inflection point (CVAI)	β-coefficient (95% CI)*	*p*-value
Marrow PDFF	<128.3	−0.0055 (−0.0064 to −0.0045)	<0.001
	≥128.3	−0.0006 (−0.0030 to 0.0018)	0.618
	Likelihood ratio test		<0.001

*Adjusted for age, time since menopause, waist circumference, body mass index, physical activity, lipid profiles, and bone mineral density.

## 4 Discussion

This study found that higher CVAI values were associated with elevated BMD in the lumbar spine, femoral neck, and total hip. These findings align with recent research indicating that postmenopausal women with osteoporosis tend to have lower VAI levels, while higher VAI positively influences lumbar spine T-scores (7). Additionally, our study observed that traditional adiposity indicators, such as waist circumference and BMI, were lower in the osteoporosis group than in the normal bone mass group. This supports previous research (7, 11, 25) and reinforces the hypothesis that mechanical loading contributes to bone formation while inhibiting bone resorption (26). Despite these associations, the relationship between obesity and BMD remains debated due to inconsistent findings across populations and assessment methods. The complexity arises from the interplay between adipocytes and osteoblasts, involving both direct mechanical loading and cellular effects. Moreover, sex-specific differences further complicate this relationship. In postmenopausal women, the association follows a U-shaped pattern, with both low and high adiposity increasing osteoporosis risk. In men over 50, however, higher adiposity is linked to a lower osteoporosis risk in a linear fashion (11). Fat distribution also plays a crucial role in determining BMD outcomes. In individuals with android-type (visceral) obesity, fat distribution correlates with BMD, whereas no such association is found in those with gynoid-type obesity (27). A study using dual-energy X-ray absorptiometry in pre- and postmenopausal females showed mixed results (28). Specifically, higher visceral adipose tissue levels in premenopausal females correlate with weaker femoral neck strength, while increased subcutaneous fat in postmenopausal women is linked to declines in femoral BMD and strength indices (28). These findings highlight the need for further research to clarify the impact of adiposity patterns on bone health across different populations and life stages.

Bone marrow adipose tissue is integral to various biological processes, including metabolism, hematopoiesis, skeletal homeostasis, endocrine, and immune functions (29, 30). Prior studies have outlined the differences in size, fatty acid composition, and lipolysis between constitutive and regulated marrow adipose tissue. Li and his colleagues have discerned functional distinctions in marrow adipocytes between constitutive and regulated marrow adipose tissue (29). Nevertheless, our comprehension of marrow adipose tissue’s physiological and pathological roles remains limited. A crucial question for researchers studying osteoporosis is the timing of marrow adipose tissue accumulation concerning the loss of bone mass. Does marrow adipose tissue accumulation precede, coincide with, or follow the decline in bone mass? Aging has been demonstrated to be associated with an increase in marrow adipose tissue (13), which occurs simultaneously with a reduction in trabecular bone density. In this study, PDFF and BMD have a negative correlation, our data further corroborate previous cross-sectional research, consistently indicating that increased bone marrow adiposity is associated with decreased BMD and a higher prevalence of vertebral fractures (13, 15).

In the present study, we have unveiled a nuanced, L-shaped non-linear relationship between CVAI and vertebral marrow PDFF. Moreover, a threshold effect analysis was performed utilizing a two-piecewise linear regression, identifying 128.3 as the critical inflection point. Below this threshold, each one-unit increase in CVAI corresponds to a decrease of 0.0055 in marrow PDFF. Conversely, above the inflection point, each one-unit increase in CVAI is not significantly associated with a decrease in marrow PDFF. The beneficial effect of increasing CVAI levels on marrow fat content is most pronounced in individuals with significantly elevated CVAI. However, it is noteworthy that these individuals with extremely high CVAI levels tend to experience an increase in marrow PDFF, which may indicate excessive CVAI have a potential negative impact on bone health.

The L-shaped correlation between CVAI and PDFF can be elucidated through several plausible mechanisms. Weight-bearing exercises enhance bone mass and microarchitecture, indicating that the stress from excessive weight might strengthen bones (31). Consistent with this, substantial weight loss achieved through bariatric surgery often results in marrow adiposity and bone loss, a condition that can be ameliorated by regular exercise (32, 33). Mechanical regulation of bone and marrow fat involves modulating the functions of differentiated skeletal cells and guiding the lineage commitment of bone marrow stromal cells (BMSCs). BMSCs interpret mechanical signals to promote osteogenesis and inhibit adipogenesis (34). Furthermore, due to the heightened activity of the aromatase enzyme in the abundant adipose tissue, hyperestrogenemia likely plays a significant role in this effect (35).

Several mechanisms have been proposed to elucidate the detrimental effects of excessive obesity on bone health. One key factor is the significant alteration in both the qualitative and quantitative characteristics of bone marrow adipocytes in obesity. At the molecular level, these alterations interfere with the normal regulation of hematopoiesis, leading to an imbalance in bone remodeling (36). Research suggests that the expansion of visceral fat is closely linked to increased adipocyte formation within the bone marrow microenvironment (37, 38). This shift is driven by the reprogramming of BMSCs, which increasingly differentiate into adipocytic progenitors at the expense of osteoblasts. As a result, excessive fat accumulation within the marrow replaces osteogenic cells, contributing to decreased bone mass and quality. These structural and functional changes ultimately heighten skeletal fragility with aging (36, 39). Beyond cellular differentiation, obesity is also characterized as a state of chronic low-grade inflammation, marked by elevated levels of adipokines and pro-inflammatory cytokines such as interleukin-6 and receptor activator of nuclear factor-kappa B ligand. These inflammatory dediators further exacerbate bone loss by both bone resorption and marrow adiposity (36, 40, 41). Additionally, obesity-induced metabolic dysregulation accelerates osteoblast senescence, as evidenced by increased expression of metabolic genes associated with glycolysis and oxidoreductase activity. This hypermetabolic state places further strain on bone homeostasis, impairing its regenerative capacity (39, 42). Perhaps most crucially, genetic factors also play a pivotal role. Notably, the deletion of the FTO gene in osteoblasts leads to excessive marrow fat accumulation, highlighting FTO’s essential function in mesenchymal cell lineage commitent (43). Collectively, these findings underscore the multifaceted impact of obesity on bone integrity, driven by altered cellular differentiation, chronic inflammation, metabolic stress, and genetic regulation.

The substantial sample size for PDFF measurements constitutes a significant strength of our study. Nevertheless, it’s essential to acknowledge certain limitations. Firstly, the causal relationship between CVAI and PDFF is challenging to establish due to limitations inherent in cross-sectional observation design. The observed correlations may stem from bidirectional influences, thus complicating causal inference. To better understand the underlying mechanisms linking CVAI and marrow PDFF, further investigation through high-quality prospective studies is warranted. Second, the research was meticulously adjusted for various confounders, thereby ensuring the reliability and stability of the findings. Even after accounting for several potential confounders, the study cannot completely rule out the influence of certain unknown variables. Finally, the findings may not be generalizable to premenopausal women or men, as the study population was restricted to postmenopausal women, potentially limiting their representativeness in the general population.

## 5 Conclusion

Our findings reveal a nonlinear L-shaped relationship between CVAI and marrow PDFF in the lumbar spine, suggesting that maintaining a moderate CVAI level may optimize the CVAI/PDFF balance while preserving skeletal integrity in postmenopausal women. In contrast, excessive visceral fat may exert detrimental effects. CVAI could serve a valuable tool for clinical assessing skeletal health in this population. Further research is needed to elucidate the bidirectional relationship between CVAI and PDFF, explore its role in skeletal remodeling, and determine the impact threshold.

## Data Availability

The original contributions presented in the study are included in the article/supplementary material. Further inquiries can be directed to the corresponding authors.
